# Screening of universal DNA barcodes for identifying grass species of Gramineae

**DOI:** 10.3389/fpls.2022.998863

**Published:** 2022-09-07

**Authors:** Jianli Wang, Zhenfei Yan, Peng Zhong, Zhongbao Shen, Guofeng Yang, Lichao Ma

**Affiliations:** ^1^Pratacultural Science Institute, Heilongjiang Academy of Agricultural Sciences, Harbin, China; ^2^College of Grassland Science, Qingdao Agricultural University, Qingdao, China; ^3^Branch of Animal Husbandry and Veterinary, Heilongjiang Academy of Agricultural Sciences, Qiqihar, China

**Keywords:** DNA barcoding, haplotype, *rbcL*, *matK*, *trnL-F*, *ITS*

## Abstract

There is currently international interest in applying DNA barcoding as a tool for plant species discrimination and identification. In this study, we evaluated the utility of four candidate plant DNA barcoding regions [*rbcL*, *matK*, *trnL*-*F*, and internal transcribed spacer (*ITS*)] in seven genera of Gramineae including *Agropyron*, *Bromus*, *Elymus*, *Elytrigia*, *Festuca*, *Leymus*, and *Lolium*. Fourteen accessions were analyzed, and *matK* and *ITS* showed the highest species, subspecies, and variety discriminatory power, each resolving 11 accessions. Species discrimination using *rbcL* and *trnL*-*F* was lower, resolving 7 and 8 accessions, respectively. Subspecies and variety discrimination using *rbcL* and *trnL*-*F* could not identify 4 accessions of *Agropyron*. A technical system can be established using the proposed DNA barcode to rapidly and reliably identify the seven genera of Gramineae. This study serves as a “useful reference” for identifying the genetic diversity of grass germplasm resources. DNA barcoding can be utilized to uncover the relatives of different species within the same family or between different families. It can also be used to determine the related groups of important herbage, turfgrass, and crops and provide crucial background information for discovering excellent genes and improving existing crop varieties.

## Introduction

Canadian taxonomist Paul Hebert first proposed the concept of DNA barcoding in 2003 (Hebert et al., 2003a). It involves using one or several standard and universal DNA fragments of the genome to identify species. Because of its rapid, simple, and accurate features, DNA barcoding has been adopted worldwide to facilitate DNA recognition and species identification (Hebert et al., 2003b; Kress et al., 2005; Hollingsworth et al., 2011; Miller, 2011). Species identification is an important foundation in taxonomy, diversity management, conservation biology, and other fields. The traditional species identification methods mainly relied on morphological characterization. However, morphological identification of species is time-consuming, laborious, and prone to error. Thus, the emergence of DNA barcoding technology was a breakthrough in species identification as it can be used to precisely identify an organism at the species level (Savolainen et al., 2005).

Research on DNA barcoding for animals has seen tremendous progress since its inception. Paul Hebert utilized mitochondrial gene *COI* to characterize 11 animal phylum and identified 13,320 species based on sequence analysis. Thus, *COI* gene was selected as the general DNA barcode for identifying animal species. So far, several new animal species have been identified using the *COI* barcode, including butterflies (*Astraptes fulgerator*) (Hebert et al., 2004), *Mactra* spp. (Chetoui et al., 2022), and *Protaphorura* spp. (Sun et al., 2017). Notably, mitochondrial genes evolve at a relatively slow rate in land plants and thus are not suitable for DNA barcoding in plants. Several studies have been conducted to find ideal DNA barcodes from chloroplast and nuclear genomes of plants (Cho et al., 2004; Chase et al., 2005). At the Third International Conference on DNA Barcoding held in Mexico in 2009, participants agreed that the chloroplast genome fragments *rbcL* and *matK* would serve as the core barcode for plant DNA barcoding, and the chloroplast genome fragment *trnH*-*psbA* and nuclear gene fragment *ITS* would serve as the supplementary barcode for plant DNA barcoding.

Plant species identification is the basis of botanical research and application. In plant taxonomy, applying plant DNA barcoding can aid in the identification of some cryptic species, as well as new species (Besse et al., 2021). Liu et al. (2011) analyzed Taxus from Eurasia using four chloroplast gene fragments and one nuclear gene fragment and identified 11 species and four new taxa. DNA barcoding has also been extensively applied to identify plant germplasm resources. For example, three DNA barcodes were used to identify six species and seven easily confused plants of the genus *Sabia*, and the sequence difference rate between the *Sabia* species and the easily confused plants was as high as 24.5% (Sui et al., 2011). For instance, by analyzing the DNA barcodes of 274 plant species belonging to 87 genera, 77 plant species were found to be misidentified (China Plant BOL Group et al., 2011).

Grassland is an important green ecological barrier on China’s land, and a vital source of livelihood for farmers and herders. It is also a primary foundation for high-quality development of pastoral areas, which occupies the largest area in the northern grassland, accounting for 40.72% of the country’s total grassland area (Dong et al., 2015). Gramineae grasses are the dominant and constructive species in China’s northern grasslands. The grasses are rich in genetic information, but with the enhancement of human activities, their genetic diversity has been gradually declining; therefore, protecting the grass germplasm resources is essential (Liu et al., 2021). Species identification is the prerequisite and basis for the protection of grass germplasm resources. DNA barcoding is a potential and effective method for identification and it gets rid of the obstacle that traditional morphological identification methods rely on long-term experience (Yang et al., 2022). The application of DNA barcoding to the identification of grass species will be an innovation in the methodology of grass resource identification. Thus, DNA barcoding can be utilized for grass germplasm identification, which is critical for the protection of the diversity of grass germplasm resources. In this study, we established a DNA barcode database and uncovered the genetic relationship of 14 accessions of gramineous grasses. The aim of the present study was to determine the best DNA barcode sequences for grass accessions that are common in China’s northern grasslands and our findings provide a “useful reference” for identifying genetic diversity of grass germplasm resources.

## Materials and methods

### Plant material and DNA extraction

**FIGURE 1:**
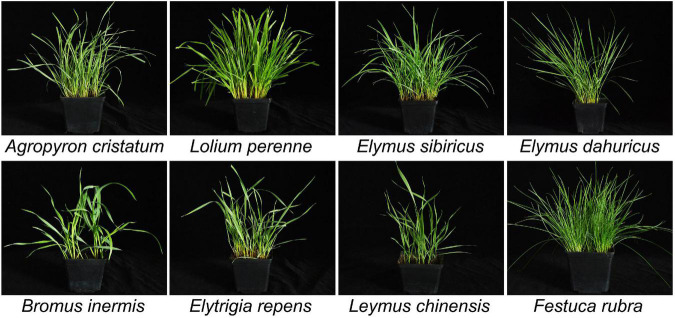
The seven genera of Gramineae.

Seeds of 14 accessions of gramineous forage grass used in this study were provided by the Pratacultural Science Institute, Heilongjiang academy of agricultural sciences, Harbin, China (Table 1). The plant samples were grown in the greenhouse under 16 h of light (390 μE m^–2^ S^–1^) and 8 h of darkness per day at 25°C (Figure 1). Four weeks later, the shoots of three plants (representing one sample) were harvested. Subsequently, total genomic DNA was extracted from each sample using the CTAB method (Doyle, 1987). Four pairs of primers were designed using DNAMAN, according to the sequences of three chloroplast genes (*matK*, *rbcL*, and *trnL*-*F*) and one nuclear region (*ITS*) of *Agropyron*, *Bromus*, *Elymus*, *Elytrigia*, *Festuca*, *Leymus*, and *Lolium*.

**TABLE 1:** Details of the 14 grass accessions used in this study.

Genera	Species	Cultivar	Location
*Agropyron*	*Agropyron cristatum var. cristatum*	—	Pratacultural Science Institute, Heilongjiang Academy of Agricultural Sciences, Harbin, China
	*Agropyron cristatum var. pectiniforme*	—	
	*Agropyron mongolicum*	—	
	*Agropyron desertorum*	—	
*Bromus*	*Bromus inermis*	—	
*Elymus*	*Elymus dahuricus*	—	
	*Elymus sibiricus*	—	
*Elytrigia*	*Elytrigia repens*	—	
*Festuca*	*Festuca rubra*	—	
*Leymus*	*Leymus chinensis*	—	
*Lolium*	*Lolium perenne*	Medalist Gold	
		Pickwick	
		Taya	
		Ascend	

### DNA barcode amplification and sequencing

The DNA sequences of the chloroplast and nuclear regions of the various grasses were obtained from the Gene Bank (Table 2). The primers were synthesized by Sangon Biotech (Shanghai) Co., Ltd. Polymerase chain reactions were then conducted in a 25-μL tube containing 1 μL genomic DNA (100 ng/μL), 1 μL of each primer (10 μmol/μL), 10 μL Takara Taq DNA polymerase master mix and water to a final volume of 20 μL. *ITS* gene was amplified under the PCR conditions of 95°C for 3 min (initial denaturation), followed by 30 cycles of denaturation at 95°C for 30 s, annealing at 55°C for 15 s, and extension at 72°C for 1 min, and a final extension at 72°C for 10 min. Meanwhile, the three chloroplast genes were amplified at 95°C for 3 min, then 30 cycles at 95°C for 30 s, annealing at 53°C for 15 s, extension at 72°C for 30 s, and a final extension step at 72°C for 5 min (Di et al., 2015). Subsequently, sequencing reactions were performed by Sangon Biotech (Shanghai) Co., Ltd.

**TABLE 2:** Primer sequences.

Primer	Sequence
*ITS*_F	GTCGTAACAAGGTTTCCGTAGG
*ITS*_R	TCCGCTTATTTATATGCTTAAA
*rbcL*_F	CCGCCTCATGGTATCCAAGTTGAAAG
*rbcL*_R	ATTTCGCGTTCCCCTTCTAACTTACC
*matK*_F	GGAACGAATCCACTTTTC
*matK*_R	GCTTTTGATAAGTATCC
*trnL-F*_F	TAATAAACACGTATAGATACTG
*trnL-F*_R	TCCTTTGTGAAAGAGTAGAATG

### Genetic diversity analysis and species delimitation

Haplotype analysis of the 11 species was performed by comparing the sequence matrices of the inland, coastal, and total samples using MEGA X to obtain a K2P genetic distance matrix (Kumar et al., 2018). Haplotype analysis was performed using DNAsp v6.12.03 (Rozas et al., 2017) for the sequences of *ITS*, *mat*K, *rbc*L, and *trn*L-F, respectively. The number of haplotypes showed included *ITS*, *mat*K, *rbc*L, and *trn*L-F. The species delimitation was based on the sequence length and haplotype of DNA barcodes.

### Evolutionary relationships of taxa

Evolutionary analysis of the plants belonging to seven genera of Gramineae was conducted using MEGA X. Sequence alignment was initially performed using Clustal W (Kumar et al., 2018). The evolutionary history was inferred using the Neighbor-Joining method (Saitou and Nei, 1987). The percentage of replicate trees in which the associated taxa clustered together in the bootstrap test (1,000 replicates) are shown next to the branches (Felsenstein, 1985). The evolutionary distances were computed using the number of differences method (Nei and Kumar, 2000) and expressed as the number of base differences per sequence. The rate variation among sites was modeled with a gamma distribution (shape parameter = 1). The proportion of sites where at least 1 unambiguous base is present in at least 1 sequence for each descendent clade is shown next to each internal node in the tree. All ambiguous positions were removed for each sequence pair (pairwise deletion option). The evolutionary analyses involved 8, 8, and 11 nucleotide sequences, as well as 706, 1,479, and 2,201 positions in the *ITS*, cpDNAs, and 4-DNA dataset, respectively.

## Results

### Sequencing and haplotype analysis

The primer sequences of *ITS*, *matK*, *trnL*-*F*, and *rbcL* were designed to allow amplification within the target regions and did not need any modification (Table 2). A total of 56 sequences were obtained from the 14 processed specimens (14 from each target gene). The size of the sequences ranged from 682 to 701 bp for *ITS*, 395 to 408 bp for *matK*, 444 to 473 bp for *trnL*-*F*, and 572 bp for *rbcL* (Tables 3–5). Haplotype analysis showed that 8 haplotypes were included in *ITS*, *matK*, and *rbcL* of 8 grass genera, and 7 haplotypes were included in *trnL*-*F* of 8 grass genera (Table 3). Between different species or subspecies of the same genus, 4 haplotypes were included in *ITS* and *matK*, 1 haplotype was included in *rbcL*, and 2 haplotypes were included in *trnL*-*F* (Table 4). Between the different varieties of *Lolium perenne*, 1 haplotype was included in *ITS*, *matK*, *rbcL*, and *trnL*-*F* (Table 5).

**TABLE 3:** Database of DNA barcoding for eight species grasses.

Species	Length (bp)					Haplotype				DNA barcoding
	*ITS*	*matK*	*rbcL*	*trnL-F*	4-DNA	*ITS*	*matK*	*rbcL*	*trnL-F*	
*Agropyron cristatum*	697	395	572	463	2127	H1^A^	H2^A^	H3^A^	H4^A^	L^2127^H1^A^H2*^A^*H3^A^H4^A^
*Bromus inermis*	696	408	572	473	2149	H1^B^	H2^B^	H3^B^	H4^B^	L^2149^H1^B^H2^B^H3^B^H4^B^
*Elymus dahuricus*	699	408	572	473	2152	H1^C^	H2^C^	H3^C^	H4^C^	L^2152^H1^C^H2^C^H3^C^H4^C^
*Elymus sibiricus*	701	408	572	470	2151	H1^D^	H2^D^	H3^D^	H4^C^	L^2151^H1^D^H2^D^H3^D^H4^C^
*Elytrigia repens*	699	408	572	470	2149	H1^E^	H2^E^	H3^D^	H4^D^	L^2149^H1^E^H2^E^H3^D^H4^D^
*Festuca rubra*	695	408	572	444	2119	H1^F^	H2^F^	H3^E^	H4^E^	L^2119^H1^F^H2^F^H3^E^H4^E^
*Leymus chinensis*	697	408	572	455	2132	H1^G^	H2^G^	H3^F^	H4^F^	L^2132^H1^G^H2^G^H3^F^H4^F^
*Lolium perenne*	696	408	572	453	2129	H1^H^	H2^H^	H3^G^	H4^G^	L^2129^H1^H^H2^H^H3^G^H4^G^

**TABLE 4:** Database of DNA barcoding for four samples of *Agropyron* species.

Species	Length (bp)					Haplotype				DNA barcoding
	*ITS*	*matK*	*rbcL*	*trnL-F*	4-DNA	*ITS*	*matK*	*rbcL*	*trnL-F*	
*Agropyron cristatum var. cristatum*	697	395	572	463	2127	H1^A^	H2^A^	H3^A^	H4^A^	L^2127^H1^A^H2^A^H3^A^H4^A^
*Agropyron cristatum var. pectiniforme*	697	395	572	458	2122	H1^I^	H2^I^	H3^A^	H4^H^	L^2122^H1^I^H2^I^H3^A^H4^H^
*Agropyron mongolicum*	682	408	572	463	2125	H1^J^	H2^J^	H3^A^	H4^A^	L^2125^H1^J^H2^J^H3^A^H4^A^
*Agropyron desertorum*	696	404	572	463	2135	H1^K^	H2^K^	H3^A^	H4^H^	L^2135^H1^K^H2^K^H3^A^H4^H^

**TABLE 5:** Database of DNA barcoding for four varieties of *Lolium perenne.*

Species	Cultivar	Length (bp)					Haplotype				DNA barcoding
		*ITS*	*matK*	*rbcL*	*trnL-F*	4-DNA	*ITS*	*matK*	*rbcL*	*trnL-F*	
*Lolium perenne*	Medalist Gold	696	408	572	453	2129	H1^H^	H2^H^	H3^G^	H4^G^	L^2129^H1^H^H2^H^H3^G^H4^G^
	Pickwick	696	408	572	453	2129	H1^H^	H2^H^	H3^G^	H4^G^	L^2129^H1^H^H2^H^H3^G^H4^G^
	Taya	696	408	572	453	2129	H1^H^	H2^H^	H3^G^	H4^G^	L^2129^H1^H^H2^H^H3^G^H4^G^
	Ascend	696	408	572	453	2129	H1^H^	H2^H^	H3^G^	H4^G^	L^2129^H1^H^H2^H^H3*^G^*H4^G^

### Generating DNA barcoding database

A DNA barcoding database was generated based on the differences in base number and haplotype of 4 gene fragments of *ITS*, *matK*, *trnL*-*F*, and *rbcL* in 8 grass varieties. The results showed that DNA barcodes of different species of the same genus were different (Table 3). Also, DNA barcodes of different subspecies of the same species were different (Table 4). However, the DNA barcodes of different varieties of the same species were the same (Table 5). Notably, DNA barcoding did not reveal any differences within species, but large differences among species were observed, enabling identification.

### Evolutionary relationships of taxa

**FIGURE 2:**
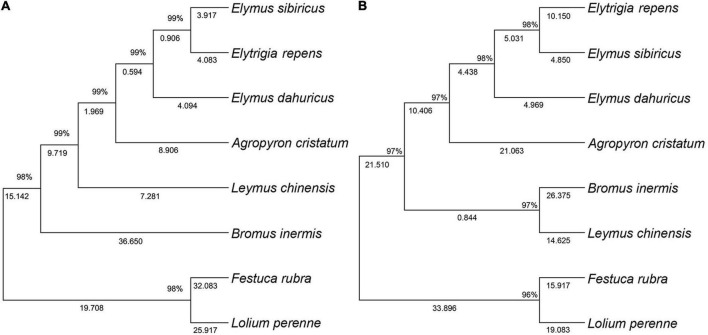
Topology resulting from Neighbor-Joining analysis of ITS and 3-cpDNA in the seven genera of Gramineae. One ITS dataset **(A)**, 3-cpDNA dataset (*matK*, *rbcL*, and *trnL*-*F*) **(B)**.

The ITS-based phylogenetic tree of the 8 grass varieties on the 706-bp alignment is shown in Figure 2A. The optimal tree with the sum of branch length being equal to the 170.97 was generated. The results showed that all the 8 grass varieties formed a monophyletic clade with a high bootstrap value. *Elymus sibiricus* and *Elytrigia repens* clustered within the same subclade, suggesting that they may have closer genetic relationships than *E. sibiricus* and *E. dahuricus*. *Festuca rubra* and *Lolium perenne* also clustered within the same subclade and were separated from the other six species, suggesting that they are more closely related in evolution.

The 3-cpDNA tree of Melilotus based on 1,479-bp of concatenated plastid sequences (*rbcL*, *matK*, and *trnL*-*F*) is shown in Figure 2B. Similar to the ITS tree for the 8 grass varieties, the 3-cpDNA tree indicated that all the 8 grass varieties formed a monophyletic clade with a high bootstrap value. *E. sibiricus* and *E. repens* also clustered within the same subclade, confirming that they may have closer genetic relationships than *E. sibiricus* and *E. dahuricus*. Meanwhile, *F. rubra* and *L. perenne* clustered within the same subclade with a single clade. The only difference compared to the ITS tree is a single subclade of *Bromus inermis* and *Leymus chinensis*, suggesting that the two are more closely related.

**FIGURE 3:**
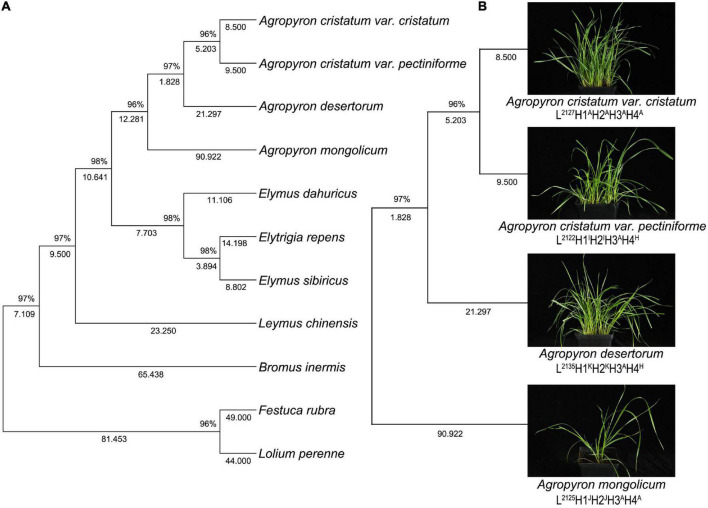
Topology resulting from Neighbor-Joining analysis of one 4-gene dataset using MEGA X. Neighbor-Joining analysis of one 4-gene dataset of 14 samples of grass belonging to Gramineae **(A)**, neighbor-Joining analysis of one 4-gene dataset in 4 samples of *Agropyron* species **(B)**.

The 4-gene tree of 11 forage species yielded 2,998 bp of four concatenated genes (*rbcL*, *matK*, *trnL*-*F*, and *ITS*) (Figure 3). The results showed that the two variants of *Agropyron* clustered in the same subclade, and the 4 species of *Agropyron* clustered together. Notably, the evolutionary relationship of the other grasses was similar to that of the ITS tree and the 3-cpDNA tree.

## Discussion

Comparison of the genetic diversity of grass germplasm resources in different regions can reveal the distribution rules of different taxa, determine the center of diversity distribution, and provide guidance for the collection and conservation of grass germplasm resources. Chloroplast genes *mat*K and *rbc*L were proposed as the candidate sequences for plant DNA barcoding by The Plant Working Group of the Consortium for the Barcode of Life (CBOL) (Kress and Erickson, 2007; CBOL Plant Working Group, 2009). However, in large scale studies, *mat*K and *rbc*L provide a discriminatory efficiency at the species level of 72 and 49.7%, respectively, and they often fail to differentiate closely related species (Kress and Erickson, 2007; Ferri et al., 2009; Li et al., 2009; China Plant BOL Group et al., 2011; Hollingsworth et al., 2011; Liu et al., 2011; Peterson et al., 2014).

*mat*K is characterized by rapid evolution and a high ability of interspecific identification, but the primer is not universal (Arca et al., 2012). Meanwhile, *rbc*L has high generality, easy amplification, and comparability, but its discriminatory efficiency at the species is not efficient. As a result, other chloroplast regions such as *trn*H-*psb*A, *trn*L, *trn*L-F (Fazekas et al., 2008; Arca et al., 2012) and the nuclear ribosomal Internal Transcribed Spacer (*ITS*) region are routinely used as supplementary barcodes alongside *mat*K and *rbc*L (Sass et al., 2007; Fazekas et al., 2008).

Diversity is key in the protection of grass germplasm resources. Evaluation of genetic diversity is crucial for the protection of grass germplasm resources and plays a guiding role in formulating the next protection objects and methods. Current genetic diversity evaluations mainly focus on understanding the genetic diversity within species by analyzing DNA markers such as RFLP (Restriction Fragment Length Polymorphism), AFLP (Amplified Fragment Length Polymorphism), RAPD (Random Amplification Polymorphic DNA), and SSR (Simple Sequence Repeats) (Brummer et al., 1995). However, a unified appraisal evaluation system has not been formulated because of the diversification of detection methods and the lack of universality of inter-species data.

Plant DNA barcodes are suitable for classification levels above species. However, significantly different populations within species can also be identified with high versatility in some taxa. Analyzing the genetic diversity of grass germplasm resources using DNA barcodes can reveal the genetic relationship between different species within a family and between different families. Moreover, DNA barcoding can be used to determine the relative groups of important pastures, turfgrass, and crops and uncover important background information for the discovery of excellent genes and superior varieties. Comparing the genetic diversity of grass germplasm resources in different regions can reveal the distribution rules of different groups, determine the diversified distribution center, and provide guidance for collecting and protecting grass germplasm resources.

Previous studies showed that the barcodes *matK* and *rbcL* had about 50% correct assignment rate (CAR) in grasses (Li et al., 2015; Loera-Sánchez et al., 2020). The low CARs for grass DNA barcodes could be due to various factors. Some grass species, such as *Poa* spp., are notoriously hard to discriminate morphologically and their phylogeny is subject to controversy. This could have resulted in misidentified reference sequences (Loera-Sánchez et al., 2020). Another factor is the high genetic similarity between some grass taxa. This may result in a higher proportion of incorrect taxonomic assignments for such grass species (Meyer and Paulay, 2005; Loera-Sánchez et al., 2020). Our results showed that the highest CAR for grasses was 100% with *matK* followed by *rbcL* (87.5%; Table 6). *ITS*, *matK*, *rbcL*, and *trnL*-*F* genes were make for good candidate for large-scale DNA barcoding of some grasses. However, further work is needed to produce reference sequences in more grass species of Gramineae.

**TABLE 6:** Species-level assignment success by barcode.

Barcode	Present study (%)	Previous studies (%)
*ITS*	100	—
*matK*	100	About 50 (Li et al., 2015; Loera-Sánchez et al., 2020)
*rbcL*	87.5	About 50 (Loera-Sánchez et al., 2020)
*trnL-F*	87.5	—
4-DNA	100	—

“—” means null value.

In this study, we utilized highly conserved universal primers to obtain ideal DNA barcoding sequences. *ITS*, *matK*, *rbcL*, and *trnL*-*F* genes were selected and used in combination to identify gramineous forages. The bases of the four gene fragments and the haplotype combination of the marker sites constituted the DNA identification code. Each forage has its own specific DNA. The identification success rate at the genus and species levels was 100%. However, this combination method could not identify different varieties of the same grass species. For example, the four perennial ryegrass varieties, including Medalist Gold, Pickwick, Taya, and Ascend have a common DNA identification code.

## Conclusion

In this study, eight forage species were identified through polymorphic locus analysis, haplotype delineation, and different haplotype combinations of marker loci. The K2P model was used to construct a phylogenetic tree, which classified the eight forage species into different clades. Combining *ITS*, *matK*, *rbcL*, and *trnL*-*F* had a significantly higher identification effect than using a single fragment. The monophyly of each species of Gramineae was verified based on auxiliary analysis of the phylogenetic tree. Our results meet the requirements of DNA barcoding to locate species in a taxonomic system (family, genus, etc.) with sufficient phylogenetic information.

## Data availability statement

The original contributions presented in this study are included in the article/supplementary material, further inquiries can be directed to the corresponding authors.

## Author contributions

JW and ZY conducted the experiments. PZ and ZS give advice and assistance in this research. GY revised the manuscript. LM designed the experiments and wrote the manuscript. All authors contributed to the article and approved the submitted version.
